# Clinical efficacy of postoperative adjuvant transcatheter arterial chemoembolization on hepatocellular carcinoma

**DOI:** 10.1186/s12957-016-0855-z

**Published:** 2016-04-02

**Authors:** Chen Liu, Li Sun, Jingchao Xu, Yongfu Zhao

**Affiliations:** Department of General Surgery, The Second Affiliated Hospital of Dalian Medical University, Dalian, 116027 Liaoning Province China; Operating Room, The Second Affiliated Hospital of Dalian Medical University, Dalian, 116027 Liaoning Province China

**Keywords:** Hepatocellular carcinoma, Hepatectomy, Adjuvant, Transcatheter arterial chemoembolization, Chronic hepatitis B

## Abstract

**Background:**

The aim of this study was to evaluate the clinical efficacy of postoperative adjuvant transcatheter arterial chemoembolization (TACE) on hepatocellular carcinoma (HCC).

**Methods:**

Data from 117 patients with HCC who underwent hepatectomy between December 2010 and February 2014 were retrospectively reviewed. In total, 55 patients underwent surgical resection only (group A), and 62 patients underwent surgical resection with adjuvant TACE (group B). The perioperative clinical indicators, postoperative sequential treatment, and follow-up were compared between the two groups of patients. The Kaplan-Meier method was used to compare survival between the groups, and prognostic factors were evaluated by a Cox proportional hazard model.

**Results:**

The two groups showed no significant difference in age, gender, preoperative A-fetoprotein (AFP) values, preoperative Child-Pugh score, hepatitis B virus(HBV) DNA levels, duration of surgery, hepatectomy technique, albumin values 1-week postoperative, postoperative complications, duration of postoperative hospital stay, cirrhosis, tumor size, tumor differentiation, tumor encapsulation, satellite nodules, or microvascular infiltration. Cox regression analysis revealed that tumor size, satellite nodules, and microvascular infiltration were significantly independent prognostic factors (*P* = 0.001, 0.002, and 0.001). Of the 117 patients, the 1-, 2-, and 3-year disease-free survival rates were 64.5, 50.0, and 41.9 %, respectively, for group B (62 patients) and 45.5, 36.4, and 30.9 %, respectively, for group A (55 patients). Although improving trends of disease-free survival were observed in the adjuvant TACE group, there was a significant difference in postoperative 1-year survival between the two groups (*P* = 0.04) but no significant difference in postoperative 2- and 3-year survival. In patients with tumor size >5 cm, the 1-, 2-, and 3-year disease-free survival rates were 41.7, 25.0, and 12.5 %, respectively, for group B and 11.8, 0, and 0 %, respectively, for group A. There was a significant difference in postoperative 1- and 2-year survival between the two groups (*P* = 0.04 and 0.03, respectively) but no significant difference in postoperative 3-year survival. In patients with microvascular infiltration, the 1-, 2-, and 3-year disease-free survival rates were 42.3, 26.9, and 15.4 %, respectively, for group B and 12.5, 4.2, and 0 %, respectively, for group A. There was a significant difference between the two groups (*P* = 0.02, 0.03, and 0.045, respectively). In patients with satellite nodules, the 1-, 2-, and 3-year disease-free survival rates were 50.0, 50, and 40 %, respectively, for group B and 17.6, 0, and 0 %, respectively, for group A. There was a significant difference between the two groups (*P* = 0.04, 0.01, and 0.03, respectively). In patients with tumor size ≤5 cm, without satellite nodules, or without microvascular infiltration, there was no significant difference between the two groups in the 1-, 2-, or 3-year disease-free survival rates. Of 117 patients overall, 18 (15.4 %) developed hepatitis B virus reactivation: 2 (3.6 %) patients in group A and 16 (25.8 %) patients in group B. There was a significant difference between the two groups (*P* = 0.000). Of these patients, one (1.8 %) patient in group A and five (8.1 %) patients in group B developed hepatitis due to hepatitis B virus reactivation. There was a significant difference between the two groups (*P* = 0.000).

**Conclusions:**

Postoperative adjuvant TACE can improve the 1-year disease-free survival rate of HCC patients. Postoperative adjuvant TACE may improve 2- and 3-year disease-free survival rates, but no statistical significance was found. For patients with tumor size >5 cm, postoperative adjuvant TACE can improve 1- and 2-year disease-free survival rates, and postoperative adjuvant TACE may improve the 3-year disease-free survival rate. For HCC patients with tumor size ≤5 cm, postoperative adjuvant TACE may improve the 1-, 2-, and 3-year disease-free survival rates, but no statistical significance was found. For patients with microvascular infiltration or satellite nodules, postoperative adjuvant TACE can improve the 1-, 2-, and 3-year disease-free survival rates. For patients without microvascular infiltration or without satellite nodules, postoperative adjuvant TACE cannot improve 1-, 2-, or 3-year disease-free survival rates. For patients with tumor size >5 cm with microvascular infiltration or with satellite nodules, postoperative adjuvant TACE was suggested. Hepatitis B virus reactivation can occur in patients with postoperative adjuvant TACE; thus, antiviral treatment was suggested for these patients.

## Background

Hepatocellular carcinoma (HCC), which is the sixth most common cancer and the third most common cause of cancer death, is the most common primary malignancy of the liver [1]. In China, nearly 90 % of HCC patients with chronic hepatitis B develop HCC through the “chronic hepatitis B-hepatitis, cirrhosis-primary hepatocellular carcinoma” trilogy. Radical hepatectomy is the most effective treatment for HCC, but there is a higher recurrence rate. The postoperative 5-year recurrence rate is 70–80 %, and the postoperative 5-year survival rate is 30–50 %.

**Fig. 1:**
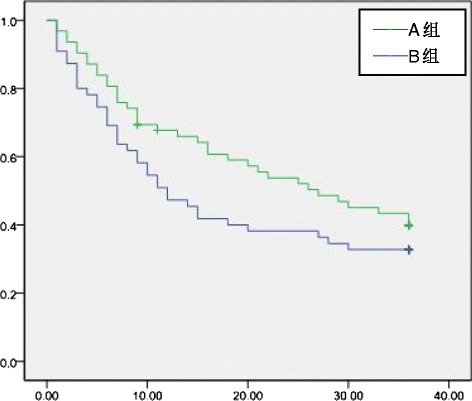
Tumor-free survival of the adjuvant TACE group and the control group

**Fig. 2:**
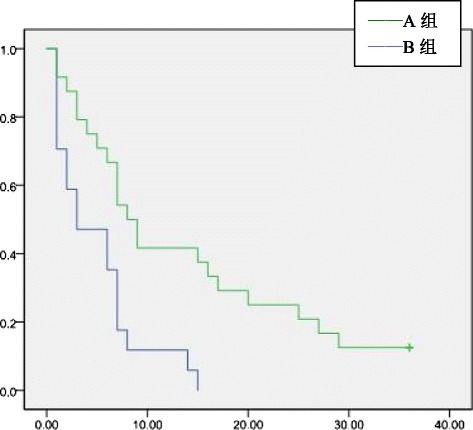
Tumor-free survival rates of patients with tumor diameter >5 cm

**Fig. 3:**
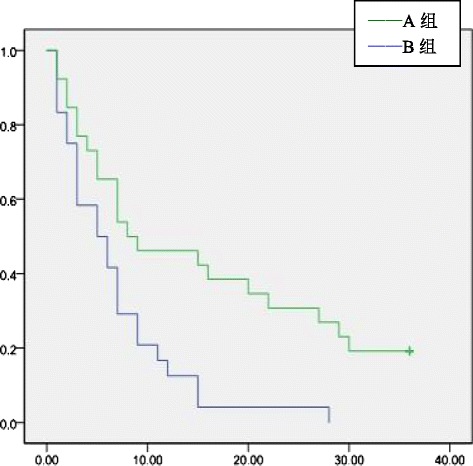
Tumor-free survival rates of patients with microvascular infiltration

**Fig. 4:**
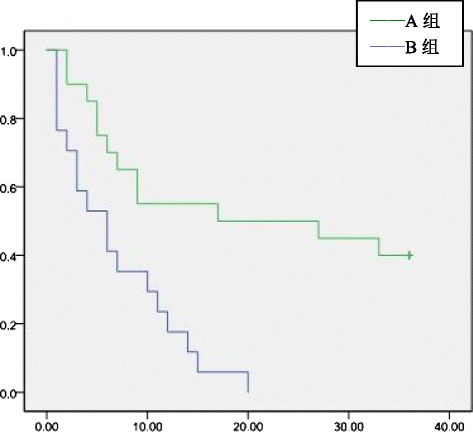
Tumor-free survival rate satellite nodules

In 1976, Goldstein first reported using transcatheter arterial chemoembolization (TACE) to treat HCC, and since then, TACE has been recognized as the preferred non-surgical method to treat HCC. Adjuvant TACE, as one of the measures to prevent the recurrence of HCC after surgery, is now widely used clinically and has accumulated substantial data, but its effects are still controversial and difficult to determine. This study analyzed the clinical data of 117 HCC patients with chronic hepatitis B who were treated from December 2010 to February 2014 in our hospital to explore the clinical efficacy of adjuvant TACE for postoperative HCC patients.

## Methods

### General information

This study included 117 patients with HCC who underwent radical hepatectomy. The entry criteria included the following: (1) all of the tumor lesions were removed, which was judged by the surgeon’s gross inspection; (2) no lymph node involvement; and (3) no distant metastasis. All of the patients were diagnosed with HCC according to the histological findings, and we excluded the cutting-edge cases of cancer tissue residue. Group A was composed of 55 patients who underwent simple radical resection: 45 males and 10 females, aged 31–73 years, with a mean age of 53.8 ± 10.5 years. Group B included 62 cases of radical resection combined with sequential TACE: 49 males and 13 females, aged 29–81 years, with a mean age of 56.4 ± 11.7 years. For the use of these clinical materials for research purposes, prior consents from the patients and approval from the Ethics Committees of The Second Affiliated Hospital of Dalian Medical University were obtained, and all the procedures have been performed in compliance with the Helsinki Declaration.

### Preoperative evaluation

In addition to routine examination, the A-fetoprotein (AFP) value, hepatitis B virus DNA value, Child-Pugh score, abdominal B ultrasound, and CT were determined.

### Adjuvant TACE

For group B patients, hepatic arteriography plus infusion chemotherapy and/or TACE was carried out conventionally at postoperative 1, 3, 6, and 12 months. Using the Seldinger method, the catheter was inserted into one side of the hepatic artery or the tumor-feeding artery. If no recurrent tumors were present, only hepatic artery infusion chemotherapy was performed: 40–60 mg/m^2^ epirubicin, 100 mg/m^2^ oxaliplatin, and 1000 mg fluorouracil. For recurrent tumors, the microcatheter or nanocatheter was inserted into the recurrent tumor blood vessels, followed by chemotherapy reperfusion combined with iodized oil emulsifier (20 mg epirubicin emulsified with 3–30 ml 30 % iodized oil suspension) embolization for each recurrent tumor. When the small branch of the portal vein appeared, the lipiodol injection was stopped.

### Definition

HBV reactivation was defined as a 10-fold or more increase in HBV DNA levels compared with baseline or the appearance of HBV DNA from an undetectable level at baseline and post-TACE HBV DNA of more than 200 IU/mL. HBV downregulation was defined as a 10-fold or more decrease in HBV DNA levels compared with baseline or the disappearance of HBV DNA from a detectable level at baseline and a baseline HBV DNA before TACE of 200 IU/mL. Patients that did not fall into either of these categories were defined as having a “stable status”. Hepatitis was defined as a threefold or more increase in serum ALT levels above the upper limit of normal (normal <40 IU/L) or an absolute increase in ALT to more than 100 IU/L. Icteric hepatitis was defined by a serum T-bil level that exceeded twice the reference range (normal <20.5 mmol/L) in the presence of hepatitis. Exacerbation of liver damage included both hepatitis and icteric hepatitis, as defined above.

### Follow-up

Ultrasonography was performed and AFP and hepatitis B virus DNA values were estimated every 2 to 3 months during the first year after hepatectomy and every 3–6 months thereafter. If tumor recurrence was suspected, visceral ultrasonography, computed tomography (CT), or magnetic resonance imaging (MRI) was performed. The follow-up time was 3 years or the day of surgery to the time of death, and survival time was calculated on a monthly basis.

### Statistical analysis

SPSS 17.0 statistical software was used for analysis. The significance of differences in clinical and pathological characteristics between groups was examined with the Chi-square test and Student’s *t* test. Cumulative survival rates were obtained by the Kaplan-Meier method. Cumulative survival comparison between groups was performed with a log-rank test. Multivariate analysis for independent prognostic factors was performed by a Cox proportional hazards model. *P* values <0.05 were considered to be statistically significant.

## Results

### Clinical and pathological characteristics of adjuvant TACE and control groups

The clinical and pathological characteristics of patients in the adjuvant TACE and control groups are summarized in Table 1. There were no significant differences in the age, Child-Pugh class, or AFP level between the two groups. Postoperative complications included the following: pleural effusion (group A: four cases, group B: seven cases); perihepatic effusion (group A: two cases, group B: five cases); liver abscess (group A: two cases, group B: three cases), cholangitis (group A: two cases, group B: one cases); and wound infection or liquid (group A: two cases, group B: five cases).

**Table 1:** Clinical and pathological characteristics of the adjuvant TACE and control groups

	Group A (55 cases)	Group B (62 cases)	*P* value
Age	53.8 ± 10.50	56.4 ± 11.7	0.21
Gender (male/female)	45/10	49/13	0.71
AFP level (ng/ml)	289.3 ± 406.1	171.8 ± 335.4	0.09
Child-Pugh class	5.21 ± 0.46	5.37 ± 0.73	0.18
A	54	59	0.37
B	1	3	
Hepatitis B virus DNA value (10^3^ copies/ml)	294.9 ± 601.1	262.3 ± 1011.9	0.83
Surgical resection			0.99
Hemihepatectomy	10	11	
Segment resection	31	35	
Wedge resection	14	16	
Differentiation grade			0.88
Well	10	13	
Middle	30	31	
Poor	15	18	
1-week-after albumin levels (g/L)	36.4 ± 3.9	36.2 ± 4.1	0.85
Complications	14 (55)	21 (62)	0.321
Average length of stay (days)	12.5 ± 5.7	14.3 ± 5.4	0.08
Liver cirrhosis	47 (55)	53 (62)	0.99
Maximum tumor diameter (cm)	4.9 ± 3.5	5.0 ± 3.1	0.96
>5 cm	17	24	0.34
≤5 cm	38	38	
Tumor encapsulation	36 (55)	38 (62)	0.64
Presence of satellite nodules	17 (55)	20 (62)	0.88
Microvascular invasion	24 (55)	26 (62)	0.85
Preemptive lamivudine therapy	18 (55)	22 (62)	0.75

### HCC recurrence risk factors

A total of 16 clinical indicators that can affect postoperative recurrence, including age, gender, tumor size, hepatitis B virus DNA, preoperative AFP value, Child-Pugh score, operation time, 1-week-after albumin levels, complications, average length of stay, liver resection, liver cirrhosis, tumor capsule, vascular invasion, satellite nodules, and tumor differentiation, were analyzed. Univariate analysis showed that age, Child-Pugh score, preoperative AFP values, hepatitis B virus DNA levels, tumor size, microvascular infiltration, and satellite nodules were significantly related to recurrence (*P* < 0.05) (Table 2). These seven factors were introduced into a Cox regression model for HCC cure, which showed that tumor size, microvascular infiltration, and satellite nodules were independent risk factors for postoperative tumor recurrence (*P* = 0.001, 0.002, and 0.001, respectively) (Table 3).

**Table 2:** Univariate analysis

Clinical indicators	*P* value
Age	0.000
Gender	0.179
Tumor size	0.000
Hepatitis B virus DNA value	0.000
AFP level	0.000
Child-Pugh class	0.000
1-week-after albumin levels	0.523
Complications	0.085
Average length of stay	0.134
Surgical resection	0.276
Liver cirrhosis	0.973
Tumor encapsulation	0.174
Microvascular invasion	0.000
Presence of satellite nodules	0.000
Differentiation grade	0.124

**Table 3:** Cox regression analysis

Clinical indicators	Regression coefficient	Standard error	*X* ^2^	Degrees of freedom	*P* value	RR	95 % CI
Tumor size	0.285	0.049	33.993	1	0.000	1.330	1.208–1.464
Age	0.010	0.011	0.016	1	0.900	1.000	0.979–1.424
Microvascular invasion	0.965	0.312	9.587	1	0.002	2.626	1.425–4.838
Presence of satellite nodules	0.942	0.252	14.033	1	0.000	2.566	1.567–4.202
AFP level	0.000	0.000	1.536	1	0.215	1.000	1.000–1.001
Hepatitis B virus DNA value	0.000	0.000	2.473	1	0.116	1.000	1.000–1.000
Child-Pugh class	0.085	0.224	0.146	1	0.702	1.089	0.703–1.688

### Tumor-free survival of adjuvant TACE group and control group

The 1-, 2-, and 3-year overall tumor-free survival rates of groups A and B were 45.5, 36.4, and 30.9 % and 64.5, 50.0, and 41.9 %, respectively. The tumor-free survival rate difference was statistically significant 1 year after surgery (*P* = 0.04). The difference in the tumor-free survival rate at postoperative 2 and 3 years was not statistically significant (*P* > 0.05)(Fig 1). In groups A and B, the 1-, 2-, and 3-year tumor-free survival rates of patients with tumor diameter >5 cm were 11.8, 0, and 0 % and 41.7, 25.0, and 12.5 %, respectively. In patients with tumor diameter >5 cm, the postoperative 1- and 2-year tumor-free survival rates were significantly different between groups A and B (*P* = 0.04 and 0.03, respectively), but the tumor-free survival rate at 3 years was not significantly different (*P* > 0.05)(Fig 2). For patients in groups A and B who had microvascular infiltration, the 1-, 2-, and 3-year tumor-free survival rates were 12.5, 4.2, and 0 % and 42.3, 26.9, and 15.4 %, respectively, and the difference was statistically significant (*P* = 0.02, 0.03, and 0.045, respectively)(Fig 3) . In group A and group B, patients who had satellite nodules, the 1-, 2-, and 3-year tumor-free survival rates were 17.6, 0, and 0 % and 50.0, 50.0, and 40.0 %, respectively, and the difference was statistically significant (*P* = 0.04, 0.01, and 0.03, respectively) (Fig 4). In patients who had a tumor diameter ≤5 cm, no microvascular invasion, and no satellite nodules, the 1-, 2-, and 3-year tumor-free survival rates after surgery were not significantly different between groups A and B (*P* > 0.05) (Table 4,5).

**Table 4:** Tumor-free survival rates

Clinical indicators	Group A			Group B		
	1 year	2 years	3 years	1 year	2 years	3 years
Overall	45.5 %	36.4 %	30.9 %	64.5 %	50.0 %	41.9 %
Tumor diameter > 5 cm	11.8 %	0 %	0 %	41.7 %	25.0 %	12.5 %
Tumor diameter ≤ 5 cm	60.5 %	52.6 %	44.7 %	78.9 %	65.8 %	57.9 %
With microvascular infiltration	12.5 %	4.2 %	0 %	42.3 %	26.9 %	15.4 %
Without microvascular infiltration	77.4 %	67.7 %	61.3 %	77.8 %	63.9 %	55.6 %
With satellite nodules	17.6 %	0 %	0 %	50.0 %	50.0 %	40.0 %
Without satellite nodules	63.2 %	57.9 %	50.0 %	69.0 %	47.6 %	38.1 %

**Table 5:** Tumor-free survival rates

Clinical indicators	1 year		2 years		3 years	
	*X* ^2^	*P*	*X* ^2^	*P*	*X* ^2^	*P*
Overall	4.29	0.04	2.20	0.14	1.52	0.22
Tumor diameter > 5 cm	4.29	0.04	4.98	0.03	2.23	0.13
Tumor diameter ≤5 cm	3.06	0.08	1.36	0.24	1.32	0.25
With microvascular infiltration	5.50	0.02	4.81	0.03	4.01	0.045
Without microvascular infiltration	0.001	0.97	1.11	0.74	0.23	0.64
With satellite nodules	4.22	0.04	11.6	0.01	8.68	0.03
Without satellite nodules	0.31	0.58	0.85	0.36	1.15	0.28

### Hepatitis B virus reactivation after sequential TACE

Overall, in the two groups, 18 (15.4 %) patients showed hepatitis B virus reactivation: 2 cases in group A (3.6 %) and 16 cases in group B (25.8 %). The difference was statistically significant (*P* = 0.000). There were six (5.1 %) patients with hepatitis caused by hepatitis B virus reactivation: one case in group A (1.8 %) and five cases in group B (8.1 %), and this difference was statistically significant (*P* = 0.000). There were no cases of fulminant hepatic failure due to hepatitis B virus reactivation in either group.

## Discussion

The HCC recurrence rate is 70–80 % 5 years after radical hepatectomy; in approximately 78–96 % of these patients, HCC recurrence occurred around the intrahepatic primary tumor location or in multiple intrahepatic locations [2, 3]. The largest report of resected patients comes from the Liver Cancer Study Group in Japan [4], which has reported 1-, 3-, 5-, and 10-year survival rates of 85, 64, 45, and 21 %, respectively, in 6785 cirrhotic patients treated by hepatic resection between 1988 and 1999. In approximately 2/3 of patients with tumor recurrence after 2 years, known as early recurrence, intrahepatic micrometastasis occurs and is associated with the risk factors tumor size, microvascular invasion, and satellite nodules. Tsai et al. [5] reported that 59 % of HCCs were accompanied by microscopic venous invasion; 40.5 % of patients with tumor diameter ≤2 cm had microscopic venous invasion. Identifying preoperative and intraoperative intrahepatic micrometastasis is difficult. The remaining recurrences occur after 2 years (delayed recurrences) and may correspond to “de novo” tumors in the oncogenic cirrhotic liver. The risk factors associated with delayed recurrence are presence of cirrhosis, hepatitis activity, vascular invasion, moderate or poorly differentiated HCC, and multinodularity [6]. Our univariate analysis showed that age, preoperative Child-Pugh score, preoperative AFP value, preoperative hepatitis B virus DNA, tumor size, microvascular invasion, and satellite nodules were associated with postoperative recurrence. According to the multivariate regression analysis, the independent risk factors for postoperative tumor recurrence were tumor size, microvascular infiltration, and satellite nodules (*P* = 0.001, 0.002, and 0.001, respectively). This result is consistent with results reported in the literature. Thus, intrahepatic micrometastasis and chronic hepatitis B treatment have become major issues in the prevention of HCC recurrence.

TACE is currently recognized as the preferred method of non-surgical treatment for HCC. However, there is a large controversy about the clinical efficacy of postoperative adjuvant TACE to prevent postoperative recurrence of HCC. Some scholars believe that adjuvant TACE can effectively inactivate or inhibit postoperative intrahepatic residual tumors or the proliferation of tumor tissue, reduce the postoperative recurrence rate, and prolong the survival time of patients. Tanaka K, et al. [7] analyzed the clinical data of 65 HCC patients who underwent hepatectomy with adjuvant TACE and showed that it can effectively reduce the 1-year postoperative recurrence rate. Jia ZZ, et al. [8] proved that adjuvant TACE can reduce the 1-year recurrence rate but could not reduce the long-term recurrence rate. Adjuvant TACE performed 1–2 months after surgery is recommended as a routine method to prevent recurrence. In contrast, other studies were not in agreement with such conclusions. They reported that adjuvant TACE could decrease both disease-free survival and overall survival rates. It was believed that TACE could damage hepatic and immunological function and therefore has a negative effect. In addition, some scholars believe that postoperative adjuvant TACE cannot kill all of the tumor cells and that it is accompanied by tumor necrosis, which can decrease tumor cell adhesion ability, leading to an increase in extensive transfer opportunities. Edward CS, et al. [9] reported that in 66 HCC patients who underwent hepatectomy, the 1-, 2-, and 3-year tumor-free survival rates in the adjuvant TACE group were lower than in the control group, and the extrahepatic recurrence probability was higher than in the control group. We found that overall, adjuvant TACE can improve the tumor-free survival rate at 1 year, showing a preventive effect on early tumor recurrence, and that there was an increasing trend for 2- and 3-year tumor-free survival rates in the adjuvant TACE group. Postoperative adjuvant TACE may improve the 2- and 3-year tumor-free survival rates of HCC patients.

Therefore, establishing the indications of adjuvant TACE has important clinical significance. However, there is still a lack of uniform consensus for adjuvant TACE. Many retrospective studies focused on the risk factors of HCC recurrence, such as microvascular invasion, satellite nodules, and tumor size, but their conclusions were not the same. Xi T et al. [10] reported that in 823 HCC patients who underwent hepatectomy, adjuvant TACE cannot effectively reduce the recurrence rate in patients with a tumor diameter less than 3 cm. TACE can improve tumor-free survival rates in patients with a tumor diameter of 3–10 cm, positive AFP value and vascular invasion or patients with a tumor diameter greater than 10 cm, AFP positivity, and multiple satellite nodules associated with vascular invasion. Li Ke-Wei et al. [11] reported that in 76 HCC patients who underwent hepatectomy, postoperative adjuvant TACE may improve 1-, 3-, and 5-year tumor-free and overall survival rates of HCC patients with microscopic venous invasion, but no statistical significance was found. TACE can be used as a preventative treatment but not a routine procedure for such patients. Peng BG et al. [12] reported that in 126 cases of HCC patients with portal vein tumor thrombus who underwent hepatectomy, the adjuvant TACE group had a better tumor-free survival rate relative to the control group. Multivariate regression analysis showed that tumor size, microvascular infiltration, and satellite nodules were three independent risk factors affecting tumor recurrence. We found that in patients with tumor diameter >5 cm, sequential TACE could effectively improve the 1-year and 2-year postoperative tumor-free survival rates (*P* = 0.04 and 0.03, respectively). Postoperative adjuvant TACE may improve the 3-year tumor-free survival rate, but the difference was not statistically significant (*P* > 0.05). For HCC patients with tumor size ≤5 cm, postoperative adjuvant TACE may improve 1-, 2-, and 3-year tumor-free survival rates, but no statistical significance was found. For patients with microvascular infiltration or satellite nodules, postoperative adjuvant TACE can improve the 1-, 2-, and 3-year tumor-free survival rates. For patients without microvascular infiltration or without satellite nodules, postoperative adjuvant TACE cannot improve 1-, 2-, or 3-year tumor-free survival rates. For patients with tumor size >5 cm, with microvascular infiltration or with satellite nodules, postoperative adjuvant TACE was suggested.

Regarding the timing and method for adjuvant TACE, there is no uniform opinion. Most HCC recurrent tumors appeared 2 years after surgery, so most scholars believe that adjuvant TACE after surgery should be carried out as soon as possible. The recommended schedule is 1 month postoperative. Pengfei Liu et al. [8] proposed that adjuvant TACE should be carried out within 1–2 months after surgery as a regular method of prevention. However, there is controversy regarding the number of times postoperative adjuvant TACE should be administered. Some scholars believe that if patients with liver function can tolerate it, adjuvant TACE treatment should be repeated throughout the entire recurrence peak period (1–2 years after surgery). However, other scholars believe that repeated adjuvant TACE treatment in a cirrhosis background may lead to an increase in hepatic dysfunction. Therefore, unless the liver is non-cirrhotic, adjuvant TACE should not be repeatedly implemented. Xi T et al. showed that single TACE is superior to repeated TACE [10].

In malignant tumors associated with chronic hepatitis B, especially lymphoma, systemic chemotherapy has been confirmed to cause hepatitis B virus reactivation in a large number of patients. In China, nearly 90 % of patients with HCC are associated with chronic hepatitis B. Increasing attention has been directed toward whether TACE can cause hepatitis B virus reactivation in HCC patients and the postoperative liver function and prognosis. There is no agreement by scholars in this field of research. Jeong won Jang et al. [13] found that transarterial chemo-lipiodolization can reactivate HBV, and HBeAg-positive HCC patients receiving chemo-lipiodolization should be closely monitored for HBV reactivation. Preemptive lamivudine therapy demonstrated excellent efficacy in reducing hepatitis due to HBV reactivation and hepatic morbidity during TACE. Preemptive therapy should be considered in HCC patients with an HBV DNA level higher than 10^4^ copies/mL. Further studies are needed to confirm the value of this approach in patients with low-level viremia [14]. However, Park JW et al. [15] believed that one session of TACE using doxorubicin and lipiodol does not significantly aggravate HBV hepatitis in patients with HBV-related HCC. Lao XM, et al. [16] believed that HBV DNA changes after TACE included reactivation and decreased and stable HBV DNA levels. Although HBV reactivation did not necessarily result in the exacerbation of liver damage and most HCC patients with Child-Pugh grade A and B tolerated TACE well, careful post-procedure monitoring and management is needed. Our results showed that hepatitis B virus reactivation can occur in patients undergoing postoperative adjuvant TACE; thus, antiviral treatment was suggested for these patients.

## Conclusions

Postoperative adjuvant TACE can improve the 1-year disease-free survival rate of HCC patients. Postoperative adjuvant TACE may improve 2- and 3-year disease-free survival rates, but no statistical significance was found. For patients with tumor size >5 cm, postoperative adjuvant TACE can improve 1- and 2-year disease-free survival rates, and postoperative adjuvant TACE may improve the 3-year disease-free survival rate. For HCC patients with tumor size ≤5 cm, postoperative adjuvant TACE may improve 1-, 2-, and 3-year disease-free survival rates, but no statistical significance was found. For patients with microvascular infiltration or satellite nodules, postoperative adjuvant TACE can improve 1-, 2-, and 3-year disease-free survival rates. For patients without microvascular infiltration or without satellite nodules, postoperative adjuvant TACE cannot improve 1-, 2-, or 3-year disease-free survival rates. For patients with tumor size >5 cm, with microvascular infiltration or with satellite nodules, postoperative adjuvant TACE was suggested. Hepatitis B virus reactivation can occur in patients with postoperative adjuvant TACE; thus, antiviral treatment was suggested for these patients.
